# Viral Hepatitis and Hepatocellular Carcinoma: State of the Art

**DOI:** 10.3390/pathogens10111366

**Published:** 2021-10-22

**Authors:** Toofan Datfar, Michael Doulberis, Apostolis Papaefthymiou, Ian N. Hines, Giulia Manzini

**Affiliations:** 1 Department of General and Visceral Surgery, Hospital of Aarau, 5001 Aarau, Switzerland; giulia.manzini@ksa.ch; 2 Department of Gastroenterology and Hepatology, Hospital of Aarau, 5001 Aarau, Switzerland; michael.doulberis@ksa.ch; 3 Department of Gastroenterology, University Hospital of Larissa, 41110 Larissa, Greece; appapaef@hotmail.com; 4 Department of Nutrition Science, East Carolina University, Greenville, NC 27858, USA; hinesi@ecu.edu

**Keywords:** viral hepatitis, hepatocellular carcinoma, HCC, cancer, risk factor, carcinogenesis

## Abstract

Viral hepatitis is one of the main causes leading to hepatocellular carcinoma (HCC). The continued rise in incidence of HCC suggests additional factors following infection may be involved. This review examines recent studies investigating the molecular mechanisms of chronic hepatitis and its association with hepatocarcinogenesis. Hepatitis B virus patients with genotype C display an aggressive disease course leading to HCC more than other genotypes. Furthermore, hepatitis B excretory antigen (HBeAg) seems to be a more sensitive predictive tumor marker exhibiting a six-fold higher relative risk in patients with positive HBsAg and HBeAg than those with HBsAg only. Single or combined mutations of viral genome can predict HCC development in up to 80% of patients. Several mutations in HBx-gene are related with higher HCC incidence. Overexpression of the core protein in HCV leads to hepatocellular lipid accumulation associated with oncogenesis. Reduced number and decreased functionality of natural killer cells in chronic HCV individuals dysregulate their surveillance function in tumor and viral cells resulting in HCC. Furthermore, high T-cell immunoglobulin and mucin 3 levels supress CD8+ T-cells, which lead to immunological dysregulation. Hepatitis D promotes HCC development indirectly via modifications to innate immunity, epigenetic alterations and production of reactive oxygen species with the LHDAg being the most highly associated with HCC development. Summarizing the results, HBV and HCV infection represent the most associated forms of viral hepatitis causing HCC. Further studies are warranted to further improve the prediction of high-risk patients and development of targeted therapeutics preventing the transition from hepatic inflammation–fibrosis to cancer.

## 1. Introduction

Hepatocellular carcinoma (HCC) represents the fourth most common cause of cancer-related death worldwide and is responsible for over 80% of primary liver disease [1,2]. Various risk factors for HCC development have been identified; age, gender, liver cirrhosis, non-alcoholic fatty liver disease and exposure to toxins such as aflatoxins, aristolochic acid, and tobacco, viruses consist some of the main risk factors leading to chronic liver disease (CLD) and HCC [3]. To date, five distinct viral hepatitis forms are acknowledged, with Hepatitis B Virus (HBV) and Hepatitis C Virus (HCV) displaying the strongest association with HCC development. Whereas chronic HBV infection accounts for over 50% of all HCC cases worldwide [4], the HCV infection attribution to HCC has lessened due to the revolutionary direct acting antiviral (DAA) therapies [5]. This trend does not apply to cirrhotic patients, who are at higher risks for developing HCC. Non-alcoholic steatohepatitis (NASH), as hepatic component of metabolic syndrome, represents the precursor step to liver cirrhosis and HCC. Due to rising prevalence of obesity pandemic, NASH attracted more attention in last years representing 15–20% of NASH-related HCC cases, 25–30% of which occur even in the absence of liver cirrhosis [6].

The pathophysiology of HCC is a complex multistep process. Identification of the cell of origin remains debatable if arising from a hepatic stem cell or transformation of mature hepatocytes. A plethora of studies based on mouse models have described carcinogenesis originating from mature hepatocytes [7]. Cancer-driver gene mutations observed in over 80% of HCC cases include telomerase activation via TERT mutations, viral insertions, chromosome translocation and gene amplification [8,9]. In NASH-associated HCC accumulation of fatty acids in hepatocytes is known to cause oxidative and endoplasmic stress inducing inflammation and cell damage [10]. Inflammatory–oncogenic signaling pathways include nuclear factor kappa-light-chain-enhancer of activated B cells (NF-κB) and tumor necrosis factor (TNF) leading to HCC induction [11]. The exact molecular mechanisms leading to hepatocarcinogenesis still need to be fully elucidated. In this review, we summarize the current knowledge about viral-induced HCC as well as host factors responsible for development of HCC.

## 2. Hepatitis A

HAV is a small non-enveloped virus, belonging to the Picornaviridae family [12]. The virus is predominantly transmitted through fecal-oral route, frequently from direct person-to-person contact or consumption of contaminated water or food, though blood transfusion-transmission has been reported [13,14,15,16]. HAV infection follows specific regional patterns analogous to the respective socioeconomic and sanitary conditions, thus reflecting the fecal-oral transmission route [17]. When infected, most adults develop clinical manifestations of self-limiting icteric hepatitis, whereas less than 30% of young children suffer from clinical symptoms. Extra-hepatic manifestations of this infection including damage to the heart, bone marrow, blood vessels or other tissues have also been described. Increasing age and co-existing chronic liver disease are specific risk factors for acute liver failure in 0.015–0.5% of patients, predisposing to death [18].

Considering any potential direct impact on HCC, HAV lacks chronicity and progression to cirrhosis, which are thought to be prerequisites of hepatocellular oncogenesis [19] and HAV past infection is not associated with the development of HCC [20,21]. Nevertheless, the pathophysiological background of HAV-mediated tissue damage implies a potential indirect contribution to HCC, at least in specific sub-populations [18]. Relatively, patients with acute HAV infection could consequently develop autoimmune hepatitis (AIH) [22,23]; AIH can progress to liver cirrhosis and the subsequent development of HCC [24,25]. Therefore, HAV-related AIH may also potentially indirectly contribute to liver oncogenesis.

The HAV-related tissue injury is mediated by an intense immune response, rather than a direct HAV-related toxicity, and an uncontrolled latent stimulation of the host’s defence could be the main mechanism. Several reports have concluded that the presence of the HLA-DRB1*1301 allele is associated with slow HAV clearance, persistent liver damage and autoantibodies expression among patients with hepatitis A [26]. In this regard, HLA-DRB*1301, also an indicator of AIH, may be related to the increasing prevalence of AIH associated with ongoing sporadic HAV epidemics [27,28].

Additionally, the impact of acute HAV infection in patients’ outcome is strongly dependent on the health of the liver at the time of infection. Thus, in patients with CLD, the addition of HAV infection predisposes to acute liver failure and/or cirrhosis decompensation and progress [19].

Increasing evidence supports a unique and robust (auto-)immunity against liver antigens after HAV infection in certain populations. More specifically, patients with functional defects in T cells, regulating immune responses against specific surface liver antigens, were found to be vulnerable to type I AIH development after hepatitis A infection, due to persistence of upregulated T helper cells and antibodies against autoantigens [29]. Furthermore, the activation and clonal proliferation of HAV-specific T cells results in their diffusion into the cerebrospinal fluid after recognition of central nervous system self-antigens, a process called epitope spreading [30]. Alternatively, the reported cases of post-HAV demyelinating diseases could reflect the suggested molecular mimicry pattern, where anti-viral specific T-cells recognize a common host’s epitopes, thus triggering and perpetuating autoimmune inflammation [31,32]. On the other hand, the upregulation of T regulatory cells (Treg) during acute hepatitis A, induced by bilirubin stimulated galectin9 (GAL-9)/ T cell immunoglobulin domain and mucin domain 3 (TIM-3) cascade, also suppresses the cytotoxicity of CD4+ T cells via both antiproliferative and apoptotic signals [33]. Nevertheless, the GAL-9/TIM-3 pathway downregulates normal immunity and facilitates the survival of HCC malignant cells, thus constituting a potential target of future treatments [34].

Taking together, HAV infection may exert a potential impact on HCC onset, progression and outcome, through immune dysregulation, and thus further large-scale studies are essential to illuminate in depth this field.

## 3. Hepatitis B

Hepatitis B virus (HBV) is one of the most common chronic infections and represents a global health problem. With an estimated number of 257 million infected subjects worldwide, it represents the leading cause of developing HCC worldwide [35]. The risk of acquiring hepatitis B was drastically reduced by high hygiene standards, screening of blood products and introduction of a prophylactic vaccine [36].

Epidemiological studies exhibit evidence of a causal role of chronic HBV infection and the development of a HCC. Questions remain as to the exact molecular mechanisms by which chronic HBV infection leads to HCC. Hepatitis B virus is a partially double stranded DNA-virus with at least eight major genotypes (A to H). During an infection the HBV DNA is being integrated, transcribed and translated leading to reproduction of the virus and its components causing an inflammatory cascade within the hepatocyte. Repeated inflammatory cycles can lead to chronic inflammation and cirrhosis, which is a high-risk factor for hepatocarcinogenesis [7]. Nevertheless, a striking difference of chronic HBV infection compared to rest viral hepatitides with direct cilinical significance, is the ability of HBV to cause HCC even in the absence of predisposing liver cirrhosis [7].

Chronic HBV infection represents the major etiological factor for HCC worldwide with more than one half of HCC patients being chronic carriers with an increased lifetime risk 25–37 times higher than non-infected patients [37,38]. Developing HCC due to chronic HBV infection is multifactorial, depending on viral status such as viral load, presence of liver cirrhosis, HBV genotypes, hepatitis B excretory antigen (HBeAg) serostatus and mutations of viral genome arising during chronic HBV infection. Not only responsible for progression of chronic liver disease, these parameters are related to the infection response to anti-viral therapies [39,40,41,42]. In addition, the risk of developing HCC remains high in HBsAg-negative HBV patients and those with occult infections [43,44].

In a prospective cohort study with more than 3600 patients with a mean follow-up of 11 years, it was demonstrated that the risk of HCC development was associated with high HBV DNA serum levels [42]. In another prospective study, Chen et al. underwent a follow-up for 426 HBV positive patients for 1664 person years [45]. Eleven (11)% of them had underlying liver cirrhosis. Therefore, the overall incidence of HCC in this cohort was 1052 cases per 100.000 person years. Only 8% of all HCC cases were derived from a non-cirrhotic liver. The two non-cirrhotic HBV patients were infected with genotype C HBV. High viremia and cirrhotic disease course is a high risk factor leading to a HCC even after successful antiviral therapy [7]. Although the majority of HCC cases occur in cirrhotic livers, as mentioned above, a significant fraction of HBV-related HCC occurs in the absence of liver cirrhosis [46]. The lower rate of underlying cirrhosis in HBV-related HCC compared to other etiologies argues for a more direct role of HBV in tumour process. To date, there are eight different Hepatitis B genotypes identified [47,48,49] from which some tend to be at a higher risk of developing HCC. While genotype B and C are most prevalent in Southeast Asia, case-control studies revealed a higher incidence and more aggressive course of disease for genotype C developing HCC [45,50]. These findings were independent of the presence of liver cirrhosis. One of the main factors responsible for tendency to malignancy is thought to be higher basal core promoter (BCP) mutation rates in genotype C HBV [45].

On molecular level, HBsAg-positivity is generally associated in screening and epidemiological studies. Nevertheless, several case-control studies indicate that HBeAg is a better predictive marker for HBV-related HCC with higher prevalence. The relative risk is increased six-fold among patients positive for HBsAg and HBeAg than those positive for HBsAg only [51]. Yang et al. diagnosed 111 cases of HCC in 11.893 HBV patients during a follow-up of 92.359 person years. The HCC incidence rate was 39 for both HBsAg and HBeAg negative patients, 324 among only HBsAg positive patients and 1169 among both positive for HBsAg and HBeAg. Positivity for HBeAg indicating active replication might be a predictor of hepatocarcinogenesis due to recurrent necrosis and regeneration. Increased turnover of hepatocytes increases risk of accumulation of spontaneous mutations or even may lead to direct implementation of HBV-DNA into proto-oncogenes or tumor-suppressor-genes disturbing them in their function. Representing active replication of HBV DNA, HBeAg can be a further useful marker for HBV-related HCC prediction, beyond the well-established for clinical practice HCC tumor marker α fetoprotein (AFP) [7].

A possible correlation between BCP mutation and genotype C HBV was already mentioned. Independently from genotypes B and C HBV, the so-called “T1762/A1764” mutation in the basal core promoter (BCP) region has a high prevalence with HCC development in both genotypes [52]. It can be detected up to 8 years prior to HCC diagnosis. Therefore, it can be considered one of the strongest predictive biomarkers [53]. In a meta-analysis with 3729 HCC patients, individuals with the double mutation had a five-fold higher risk of developing HCC [54]. “C1653T” in Enhancer II and “T1753V” are two other gene mutations enhancing the risk of HCC development [46]. All of these mutations alone or in combination can predict HCC development in 80% of cases [55]. Risk scores, which are based on age, gender, HBV DNA level, core promoter mutations and cirrhosis have been established to estimate the risk of HCC development less than 10 years after presentation to identify high risk patients screening of HCC [56].

**Figure 1:**
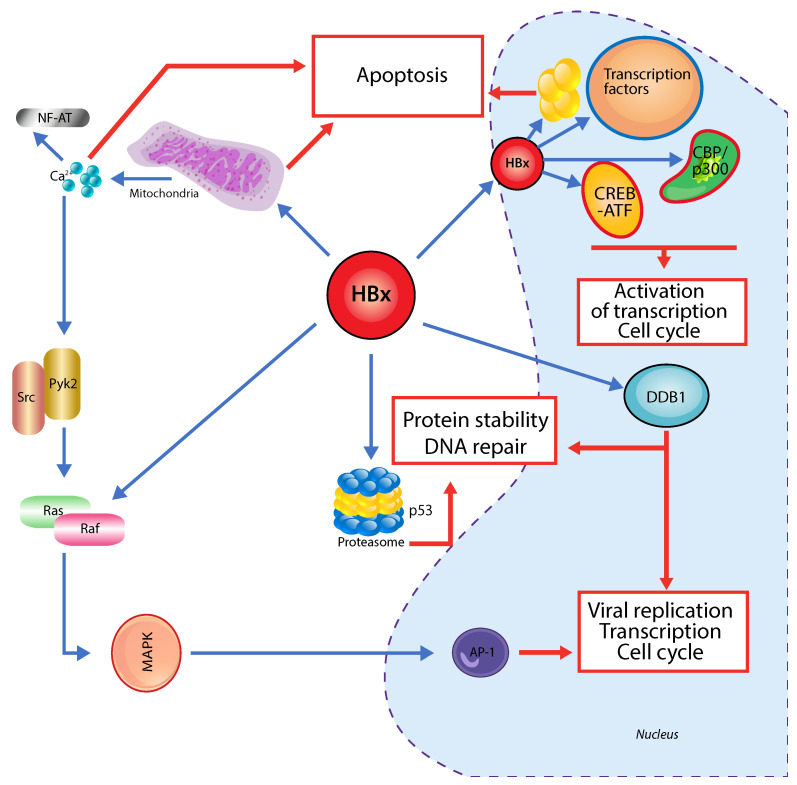
Plasmatic and intranuclear activity and protooncogentic activities of HBx-Protein in HBV. Hbx interacts with CBP/P300 affecting directly the CREB-dependent transcription. Indirectly it affects the transcription and cell cycle by acting on cellular signaling pathways such as Ras/Raf, MAPK and JAK/STAT. Apoptotic effects of Hbx are reached by affecting proteasomes, mitochondrial proteins, p53 and DDB1. HBV—Hepatitis B Virus; CBP—CREB binding protein; CREB—cAMP response element-binding protein; Ras—Rat sarcoma virus; MAPK—mitogen-activated protein kinase; JAK—Janus Kinase; STAT—Signal transducer and activator of transcription (Adapted from Neuveut C. et al. [46]).

The HBx-protein is an activator for several host cellular genes responsible for DNA repair and growth control. Among the viral products, HBx protein has been termed “viral oncoprotein” by many authors due to its pleiotropic activities on cell cycle regulation, signalling pathways and DNA-repair (Figure 1). Affecting cell cycle and cell transcription HBx takes direct effect on acetyltransferase CBP/P300-Komplex and enhances CREB/ATF affinity to cellular DNA. CREB plays not only a major role in liver metabolism but has also been implicated in hepatocarcinogenesis [46,57]. HBx has also been shown to collaborate with Ras protein in the transformation of primary human fibroblasts for a possible initiation of carcinogenesis [58]. HBx activates the mitogen-activated-protein (MAP) kinase pathway involved in hepatocarcinogenesis and regulates the tumor protein 53 gene (TP53), which is a tumor suppressor gene [59,60]. Some point mutations such as “K130M” and “V131I” in the HBx-gene are more often detected in patients with HCC than those with chronic HBV infection [61]. Furthermore, the triple mutation combination of HBx-gene “xK130M/v131I/xV5M” has been associated with four- to five-fold higher risk of HCC onset [62]. Other substitutions at positions 10, 30, 38, 88, 94 and 144 are also associated with HCC [63,64,65]. Although exact mechanisms still remain unclear, HBx plays a central role in HBV-related hepatocarcinogenesis and a possible therapeutic target for HCC suppression.

In summary, chronic HBV infection represents the main risk factor for viral induced HCC development. Chronic HBV infection is responsible for malignant disease course even in absence of liver cirrhosis. While genotype C HBV is associated with Basal core promoter mutations, there is evidence that this factor might act independently in carcinogenic pathways. Furthermore, in patients with genotype C HBV there is evidence of higher HBeAg levels, which may explain the aggressive disease course. A substantial role in carcinogenic pathways are single and combined mutations in the core promoter region as well as in the Hbx-gene, which we outlined in this review. A recent meta-analysis underlined the PreS deletion being one of the most common mutations in HBV-related carcinomas among Asians [66]. Although based on a limited number of 242 patients as mentioned by Kao et al., we sum up that viral genotype and high viremia are direct causes for hepatocarcinogenesis independent from a cirrhotic liver (Table 1).

## 4. Hepatitis C

Worldwide, more than 170 million individuals are chronically infected with HCV [67]. HCV can be divided into seven main genotypes and 67 identified subtypes with a substantial genetic divergence within driven in part by the absence of proofreading machinery in the viral RNA polymerase [67,68]. Distribution of HCV genotypes was recently evaluated with genotype 1 representing nearly 50% of the cases worldwide. Geographical differences are present within the genotypes where Type 4 shows higher prevalence in Africa and the Middle East while genotype 1 predominates in Europe and North America [68].

HCV infection results from a series of events involving translocation of blood-borne viral particles entering hepatocytes using a variety of cell membrane proteins as anchors for attachment with subsequent adjacent cell to cell transmission [69,70]. It is estimated that, in a chronically infected individual, between 30% and 50% of hepatocytes contain viral particles [71]. Parenchymal cell damage following virus infection results, in large part, from immune responses mounted against the pathogen and its specific antigens [72]. Both innate and adaptive immune cells participate, particularly hepatic macrophages in coordination with antigen specific T cell recruitment, leading to the secondary accumulation of lipid as well as activation of hepatic stellate cells, the last of which secrete transforming growth factor β and contribute and development of liver fibrosis [72]. Of note, liver fibrosis is a well-acknowledged risk factor for HCC onset [73].

The correlation between HCV infection and the development of HCC is well established [74]. Previous studies have suggested a 15 to 20-fold increase in the incidence of HCC in HCV infected patients versus HCV-negative individuals independent of genotype or subtype [75]. Unlike other liver specific viruses, the mechanisms governing this transition to carcinogenesis are not fully understood. HCV does not incorporate into the DNA directly eliminating this possibility for cancer induction [74]. Recent studies have highlighted alterations in immune cell surveillance, induction of stem-like cells by specific HCV proteins, alterations in apoptosis signaling, as well as epithelial to mesenchymal transition (EMT) as potential drivers of or negative prognostic indicators of disease progression to HCC [69,76,77,78]. It is clear that, in the majority of HCV-HCC cases, patients have progressed to liver fibrosis and cirrhosis indicating that sustained, severe disease predisposes one to cancer development [75]. The following discussion will review recent studies investigating potential pathways for HCC development in this complex, chronic disease process.

**Figure 2:**
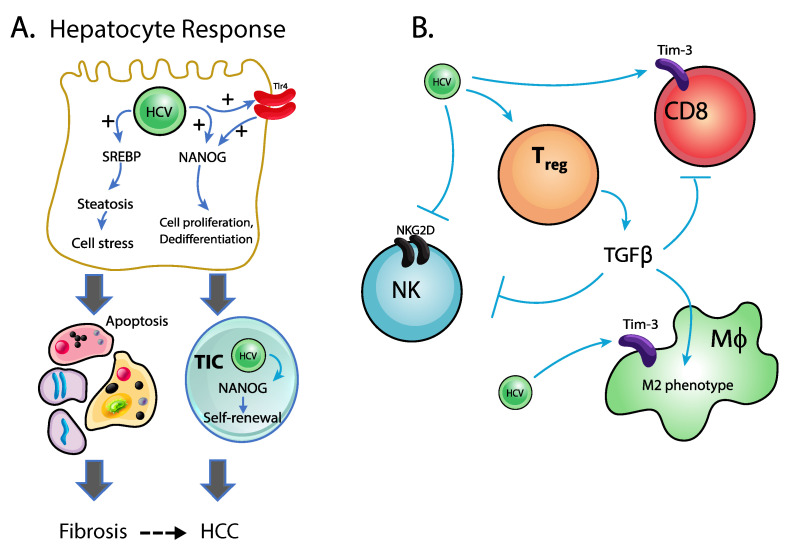
Impact of HCV on liver function and immunological response. (**A**). HCV infection leads to upregulation of SREBP (sterol receptor element binding protein) and Tlr4-augmented NANOG activation resulting in cell stress mediated apoptotic cell death and/or cellular transformation toward tumor initiating stell-like cells (TIC) whereby both hyperproliferative transformation as well as hepatocellular apoptosis driven fibrogenesis are risk factors for HCC development. (**B**). HCV infection enhances regulatory T cell recruitment and transforming growth factor b mediated suppression of NK cells and CD8+ T cells while also promoting an M2 phenotype within intrahepatic macrophages. Tim3 (T cell immunoglobulin and mucin domain-containing protein 3).

Chronic HCV infection is strongly associated with the accumulation of lipid within hepatocytes [79]. Various components of the virus have been associated with this dysregulation in lipid metabolism [80]. Specifically, early studies by Miyoshi and colleagues demonstrated that simple overexpression of the core protein in mice could promote significant hepatocellular lipid accumulation [80]. More recent work has confirmed this effect detailing the ability of the NS3a protein component to localize to the lipid droplets within hepatocytes and enhance fatty acid synthase expression through activation of SREBP-1 leading to increased lipid synthesis [81,82]. These changes in lipid accumulation alone increase oxidant stress and endoplasmic reticulum stress which have been associated independently with oncogenesis [83,84] (Figure 2). Additional work showed a core protein induced change in NANOG response, leading to formation of stem cell-like cells, termed tumor initiating stem-like cells [85,86]. It was further demonstrated that NS5a promotes increased Tlr4 expression on hepatocytes leading to endotoxin-induced NANOG expression [87]. Changes in lipid metabolism and hepatic lipid accumulation have been associated with epithelial to mesenchymal transition, promoting both fibrogenesis and potentially tumor cell initiation, again through the formation of tumor initiating stem-like cells [88]. It is clear from these studies that lipid accumulation plays an important role in HCC development and highlights the danger of secondary factors such as diet or alcohol consumption, which can enhance inflammatory signaling and promote additional lipid accumulation, as added risk factors for HCC development.

Immune cell activation is instrumental in the early stages of HCV infection [72]. Activation of dendritic cells and resident macrophages, with common representatives of the latter Kupffer cells, leads to a robust T cell-mediated response which, in a small number individuals, can eliminate the viral infection during the early stages of disease [89]. For most, however, progression to CLD occurs. Growing evidence indicates that immune cell dysfunction exists, both during the chronic stage of disease as well as during the development of HCC [90]. In chronically infected patients, viral particles activate Tlr3 on the cell surface and/or RNA helicases retinoic acid inducible gene 1 (Rig-1) within the cytoplasm to initiate a potent cytokine response predominated by interferon protein production [91]. Intriguingly, studies highlight the potential of HCV to inhibit final RNA processing and/or translation machinery limiting the interferon response effectiveness. Similarly, T cell exhaustion, particularly in CD8+ T cells, has been noted in chronic HCV infected individuals [92]. It is known that elevated levels of T-cell immunoglobulin and mucin domain 3 (Tim-3) are found on the surface of virus specific CD8+ T cells within the liver and function to suppress effector T cell function through Tim-3 - high mobility group box 1 (HMGB-1) interactions [93]. Tim-3 has widely been associated as an exhaustion marker and, within the current context, suppressive CD8+ T cells as well as inhibition of effector T cells could appear as “exhausted” rather than inhibited based on their responsiveness and cytokine/effector molecule production profiles [94]. Together, these pathways of immunological dysfunction/dysregulation may represent an important mechanism by which HCV could promote HCC development.

In a recent small study, Song et al. evaluated the immune cell phenotype within HCC tumor tissue. Their results highlighted changes in both macrophage and T cell populations as well as chemokine and cytokine patterns within tumor tissue which could alter HCC progression [77]. Specifically, this study identified an anti-inflammatory M2 macrophage population with high CCL18 production which was positively associated with HCC severity. This cell subset was further correlated with the expression of cyclic AMP response element modulator (CREM) which is known to limit IL2 promoter induction in lymphocytes and may participate in macrophage polarization/recruitment [95]. This movement toward an M2-like phenotype within the tumor associated macrophages is not unusual as solid tumors in other tissues show similar anti-inflammatory characteristics [96]. Intriguingly, in the setting of HCV infection, macrophage polarization appears to move independently of typical patterns where M1 types cells express typical M2 markers including IL10 and M2-like cells continue to produce IL12 and other M1 associated factors [97]. Alterations in macrophage polarization may be central to the altered adaptive immune responses which are necessary for both virus infected cell removal as well as tumor surveillance. Ahmed and colleagues demonstrated reduced CD8+ T cell function in HCV-infected individuals despite increased levels of anti-viral cytokines including interferon gamma and IL12 [97]. Consistently, patients with HCV and HCC exhibit increased circulating numbers of T regulatory cells, both traditional FoxP3+ and non-traditional CD25- and FoxP3-, and elevated IL10 production. Perhaps more striking and likely central to the connection between advanced stage disease (i.e., F3–F4) and HCC development is the strong positive correlation with disease stage and IL10 production [97]. IL10 is well appreciated for its ability to suppress the immune response, support T regulatory cell development and function, and generally promote tolerance to antigens within the microenvironment [98].

Tumor surveillance is the function of a number of specialized immune cells including natural killer (NK) cells [99]. Cell–cell contact between tumor cell and NK cell, mediated by a variety of receptors including NKG2D promote activation and release of proteins including perforin and granzyme b which directly destroy tumor cells [100]. Supporting this idea are several experimental studies showing increased solid tumor growth in mice lacking a functional NKG2D as well as human studies showing improved clinical outcomes in patients provided allogenic NK cells following HCC cryoablation [101]. Given their involvement in both virus and cancer cell surveillance, NK cells could be a central regulator of HCV-induced HCC. However, numerous studies have reported reduced numbers/decreased functionality of NK cells in HCC patients including those associated with HCV infection [69,89,97,102,103]. As with cytotoxic CD8 T cells discussed above, defects in NK cells within the context of HCV and HCC have been reported, particularly their apparent exhaustion and diminished numbers as the stage of disease progresses [72,104]. NK cells produce less granzyme B and perforin as well as reduce their surface expression of NKG2D further limiting their effectiveness at tumor cell clearance [105]. The mechanism for this reduction in effectiveness likely revolves around both cytokines within the extracellular matrix as well as regulatory T cell presence as has previously been observed [106]. Indeed, TGFβ levels within the tumor microenvironment are inversely correlated with the numbers and functionality of intratumor NK cells [107]. Chronic HCV infection supports ECM changes and likely promotes accumulation of regulatory cytokines such as TGFβ which could provide the barrier necessary for HCC development.

NK cells may also participate in the destruction of antigen specific T cell response where increased TIM-3 and PD-1 enhances NK cell engagement with and killing of helper T cells [108]. As discussed above, alterations to Tim-3 expression may act as a central regulator of the immune response, particularly within the tumor microenvironment and, in the current context, contribute to the reduced numbers of tumor associated lymphocytes. Intriguingly, the increased expression of Tim-3 may be related to TGFβ. Studies in macrophages revealed increased Tim-3 expression on TGFβ exposed M1 macrophages and correlated this expression with their transition to an anti-inflammatory, M2 phenotype [109]. Together, these data demonstrate the dysfunction within the immune cell compartment driven both by regulatory cell activation and increased immune cell killing which ultimately limits the liver’s ability to eliminate the virus as well as cancerous cells.

DAA therapy has proven remarkably successful as a curative agent in HCV infected individuals [110]. While somewhat dependent on viral genotype and stage of disease, combination of two protease/polymerase inhibitors leads to a 90–95% eradication of HCV infection [111]. Emerging evidence indicates, however, that elimination of viral infection may not protect the patient from additional liver related pathologies. Specifically, patients treated by means of DAAs and remain prolonged with sustained virological response (i.e., cured), still have a significantly higher incidence of HCC development than the general population [74]. Vranjkovic and colleagues have recently demonstrated that specific immune cell components, namely CD8+ T cells expressing perforin and CD107+, remain elevated up to one year after HCV curative therapy [112]. Other studies have also indicated a sustained disruption in phenotype and function of NK cells within the liver. Strunz et al. reported a reduced NK cell surface receptor repertoire in HCV infected individuals which persisted well beyond curative treatment [102]. Similar further results were found in mucosal associated invariant T cells, where both reduced numbers and functionality were observed even after successful DAA treatment [113]. Finally, Treg cell numbers remain increased following DAA treatment further limiting the overall functionality of the immune response [114]. Indeed, it appears that HCV-induced changes in key anti-tumor cell populations do not fade following virus clearance.

In summary, HCV infection promotes a wide range of changes within the liver, from lipid accumulation within hepatocytes to architectural changes associated with fibrogenesis which ultimately impact liver function. Central to these changes are oxidant stress and overall tissue damage which are known to predispose the liver to cancer development independent of HCV infection itself. Growing evidence demonstrates a wide variety of HCV associated alterations to immune cell responses, whereby recruitment, differentiation, and regulation are disrupted.

## 5. Hepatitis D

Hepatitis D virus (HDV) was first described in 1977 and represents the sole member of the Deltavirus genus [115,116,117]. HDV is regarded generally as a defective virus, which necessitates the coexistence of HBV infection, particularly HBsAg as an envelope protein, in order to support its virulence [115,118]. From epidemiological aspect, two recent meta-analyses have estimated that up to 70 million individuals may be coinfected by HDV [118]. Perpetuation of the infection with both HDV and HBV could be associated with the development of liver cirrhosis in a majority of patients compared to those patients who only have chronic HBV infection [115]. Co-infection of HBV and HDV is believed to lead to the most severe form of hepatic inflammation raising the risk of liver cirrhosis occurrence by three-fold [118].

The role of HDV infection in liver oncogenesis remains a topic of debate [119]. Despite the discrepant results regarding hepatocellular carcinoma (HCC), one study showed that genotype 1 is associated with worse clinical disease including HCC than genotype 2 [120]. Moreover, a recent large meta-analysis including 29 studies demonstrated a strong association between HCC occurrence risk and HDV infection [118]. In an Italian cohort (*n* = 299) with HDV/HBV co-infection, the annual incidence of HCC-onset was estimated to be 2.8% (follow-up for 19 years) [121]. In a further relevant study (*n* = 200), it was demonstrated that HDV-infected cirrhotic patients exhibited three times more elevated risk for development of HCC [116].

The Swiss HIV cohort reported that individuals with triple infection HBV/HDV/HIV who were followed-up for a median time of 8.7 years revealed a nine-fold and eight-fold increase in HCC risk and liver related death, respectively, [116]. Relevant conclusions were also drawn from a multicenter Spanish study which investigated a total of 118 HBV-HDV co-infected individuals for a time frame of eight years [122]. The authors reported that patients with detected HDV genome (under interferon treatment) were more likely to develop liver cirrhosis. Moreover, HCC onset was observed in 6/86 treated patients with detectable HDV RNA and in 2/32 treated patients without genome detection. A further HDV study investigated epidemiological aspects of HDV infection in an endemic Spanish area. The authors reported 11.5% seropositivity of HDV in a sample pool of HBV infected individuals (*n* = 605). Two-thirds of them were regarded as having been inoculated with the virus via intravenous administration through drug use and another considerable percentage attributed nosocomially. The majority of the aforementioned HDV patients exhibited liver cirrhosis (77%) with 16.5% also having HCC. However, a statistically significant difference for HCC onset was not observed between HDV-HBV coinfection and mono-infection HBV patients, despite the more frequent decompensated liver cirrhosis of the former group [123]. In Gambia, an area with high HCC incidence, it was demonstrated by utilizing a sensitive and specific quantitative microarray antibody capture assay, that HDV infection contributed to this elevated HCC-risk [124].

At the molecular level, since HDV does not integrate into the human genome, a direct oncogenic effect is unlikely [125]. However, HDV can indirectly promote the development of HCC via modifications of innate immune responses, stimulation of adaptive immune responses, epigenetic alterations, and/or production of reactive oxygen species (ROS) [126]. Specifically, the mentioned L-HDAg appears to play an important role in accelerating many of these mechanisms via interaction with signaling pathways involved in pro-growth/survival, apoptosis, and wound healing [127,128]. Activation of the TGF-β and AP-1 pathways by L-HDAg binding of Smad3, STAT3, and c-jun promotes epithelial-mesenchymal transition (EMT), fibrosis, and cell-transformation [127]. Augmented TGF-β signaling is a crucial effector of EMT in liver cancer progression and metastasis [129]. Likewise, HDV is able to induce oxidative stress in the endoplasmic reticulum via L-HDAg’s interaction with the enzyme NADPH oxidase (NOX)-4 [130]. Activation of the NOX4 pathway causes the release of ROS which can activate STAT3 and NF-κβ signaling involved in hepatocarcinogenesis [130]. The L-HDAg can also stimulate pro-inflammatory NF-κβ activity via stimulation of TNF-α. Likewise, the S-HDAg can directly bind to glutathione S-transferase P1 mRNA inducing downregulation in expression, augmented ROS production, and apoptosis [131]. Finally, epigenetic modifications such as histone H3 acetylation by Small and Large HDAg increases clusterin gene expression [131]. In this regard, augmented levels of clusterin and histone acetylation aid in HDV infected cell survival and are upregulated in malignant cells [132].

## 6. Hepatitis E

Hepatitis E virus (HEV) is listed among the various enteric viruses involved in food-borne outbreaks and regarded as the most common etiology of acute hepatitis world-wide [133]. The nomenclature of “E” hepatitis presumably stems from its association with epidemics as well as enteric route of transmission [133]. HEV is a single strand RNA virus that belongs to the Orthoherpesvirus genus, which is a part of the wider family of Herpesviridae. There are two main human genotypes, 1 and 2, while 3 and 4 are zoonotic and, despite infecting humans, the primary reservoir are wild boars and pigs [134]. The HEV particle lacks an envelope and its size is rather small (27–34 nm). New evidence has identified enveloped forms of HEV, which can be isolated from bloodstream during the viremia phase [133]. About 20 million individuals are estimated to be infected by HEV [115]. Incubation time varies between 2 and 10 weeks and pregnant females are at elevated risk for mortality [115,135]; HEV infection is commonly a self-limiting disorder in otherwise healthy individuals, but it can trigger a fulminant hepatitis in pregnant women where it causes 20–25% of death [136].

The clinical spectrum of HEV infection ranges from asymptomatic or acute cases to chronic hepatitis in immunocompromised patients. In patients with CLD, HEV may result in decompensation and death [137]. HEV seroprevalence in European patients with CLD varies from 3.1% in Germany to 41.4% in France [137,138]. Specifically, although the course of HEV infection is acute, in immunocompromised patients, a chronic course with development of fibrosis–cirrhosis and subsequently HCC have been reported [139]. Additionally, it was recently revealed that acute HEV superinfection in individuals with already established HBV infection accelerates disease progression and raises mortality in cirrhotic patients. The authors demonstrated that HCC risk in such populations is increased [140]. Similar results were obtained from an African study from Cameroon. Here, a high seroprevalence of anti-HEV in patients with HCC developed in a setting of chronic hepatitis B or C [141]. In China, Bai et al. reported a three-fold greater HEV seroprevalence among 103 patients with HCC than in 950 controls (31% vs. 13%) [137]. Other authors reported that, among 32 Ghanaian jaundiced HCC patients, HEV was detected in two cases alone (6%) and in nine cases in co-infected with HBV (28%) [142]. Finally, Borentain et al. defined a link to HCC with chronic hepatitis E in a 65-year-old cirrhotic patient with a history of follicular lymphoma [139].

Viewing the aforementioned data, HEV appears to join along with HBV and HCV as a possible HCC etiology in chronically infected individuals. HEV may trigger HCC, as in the case of all other viral agents through the induction of chronic hepatitis [143]. As with HCV, the HEV genome is a single-stranded positive sense RNA that does not integrate totally or partially into human DNA. Furthermore, as is assumed for HCV, HEV might initiate HCC via chronic inflammation and alterations of the cellular pathways owing to interactions between viral proteins and host factors [144]. The reason why the connection between HEV and HCC has infrequently been reported might be that chronic hepatitis E seems to be constrained to severely immunocompromised patients in developed countries [145], the reported overall small number of HEV-related chronic hepatitis and cirrhosis globally [146] and the efficacy of ribavirin therapy in patients with biopsy-confirmed chronic hepatitis E [147]. Chronic HEV infection can be treated by reducing the immunosuppressive drugs dosages or by ribavirin therapy in about 80% of patients with a 3-month treatment regimen, and with an additional 6-month course in virtually all cases of virological non-response or relapse [145]. Moreover, although liver cirrhosis develops shortly following HEV infection [145], HCC usually progresses more slowly than cirrhosis; in the case report reported by Borentain et al., the patient exhibited chronic liver cytolysis, indicated by retrospective analysis of liver cirrhosis biological tests, for 8 years [139].

## 7. Discussion

Over 90% of HCC cases occur in the setting of chronic liver disease. Cirrhosis from any etiology is the strongest risk factor for HCC [7,148], which is the leading cause of death in cirrhotic patients, with an annual incidence of 1–6% [149]. The major risk factors for HCC include chronic alcohol consumption, diabetes or obesity-related non-alcoholic steatohepatitis (NASH), and viral infection [150]. Hepatitis B Virus infection is the most prominent risk factor for HCC development, accounting for about 50% of cases worldwide and over 60% in Asia and Africa [4,7]. The risk attributed to hepatitis C virus infection has substantially decreased owing to patients achieving sustained virological response with antiviral drugs [5]. Nonetheless, patients with cirrhosis are still considered to be at high risk for HCC incidence even after HCV clearance. NASH, associated with metabolic syndrome or diabetes mellitus, is becoming the fastest growing etiology of HCC, particularly in the West [6]. Additionally, reports on mutational signatures have established aristolochic acid and tobacco as potential pathogenetic cofactors in HCC [8]. Inserting and integrating its viral DNA into the host’s genome, leading to oncogene activation, HBV remains a risk factor for HCC development even in the absence of cirrhosis [7]. HCC incidences have been decreased due to HBV vaccination programme and NA therapy avoiding a chronic course. For chronic HBV and its dynamic disease course, the clinician is challenged by regular adaptation of surveillance and treatment response [151]. Identification and therapeutic treatment of an aggressive disease course from early stage might help slow down or even prevent a cirrhotic or malignant result. Further research may highlight important pathophysiological connections between the aforementioned risk factors and a malignant course on a molecular level. Genotypic differences with higher risk to malignancies in HBV patients are mainly based on studies made in Asian countries comparing genotypes B and C HBV. Further research is needed for comparison of mutants within other genotypes and their tendency to promote carcinogenesis.

In contrast to HBV, no evidence of genotype-dependent factor is seen within HCV–HCC paradigm, although its viral clearance response might differ due to the HCV genotype [111]. It seems likely that HCV-induced changes in key anti-tumor cell populations are not corrected following virus clearance. This may explain, in part, the increases in incidence of HCC in DAA treated patients. Understanding how HCV causes such long-term changes remains an important clinical question and could help to address the development of this important secondary liver pathology. Combination of chronic tissue damage coupled with altered immune cell responses likely contributes significantly to the HCV–HCC paradigm. Further research is needed to better understand the mechanisms by which immune cells are rendered dysfunctional in this scenario. Identifying key mediators could provide important targets for therapy during and after HCV clearance to prevent HCC development.

## 8. Conclusions

Current studies were reviewed to emphasize factors responsible for HCC in viral hepatitis. Cirrhosis regardless of etiology remains the main risk factor. Molecular pathways in chronic HBV infection are well known and yet still their mechanism is not well understood. In addition, the viral genotype, numerous mutations in Basal Core promoter and HBx protein correlate with higher HCC incidences. Detection of these mutations may lead to early diagnosis and prevention of malignant courses. Co-infections with HEV or HDV accelerate disease progression and lead consequently to HCC. Viral factors in malignant pathways in chronic HCV disease are less described. The studies analysed herein focus mainly on host factors. Oxidative stress, altered immune cell response and the overall tissue damage play major roles in the HCV-HCC paradigm. While HBV/HDV co-infections lead to higher risk of cirrhosis, genotype 1 HDV had worse clinical outcome than genotype 2 including malignant course. HAV and HEV can lead to secondary diseases such as AIH or co-infections characterizing risk factors for malignant outcomes.

## 9. Future Perspective

It is clear from the preceding review that viral hepatitis represents an important predisposing factor for the development of HCC. Commonalities exist in the development of HCC following viral infection including chronic inflammation, epithelial to mesenchymal transition, and overt fibrosis and cirrhosis. Not all cases produce HCC within a given hepatitis virus type, suggesting that additional factors may be at play, including host responses and secondary pathologies including metabolic syndrome and fatty liver disease. Indeed, the strong correlation among fibrosis progression and HCC development highlights the need for additional drivers of disease progression. Moreover, HCC incidence remains elevated following therapeutic clearance of virus further implicating additional factors in its occurrence. Indeed, emerging evidence indicates prolonged alterations in regulatory immune cell processes which may enhance the initial inflammatory response as well as impair secondary surveillance pathways critical for clearance of altered parenchymal cells within this inflammatory microenvironment both acutely as well as chronically. Future studies aimed at understanding the impact of various virus types on the host immune cell response may provide new insights into HCC occurence in these patients as well as new therapeutic targets to limit or prevent this disease progression.

## Figures and Tables

**Table 1:** Viral factors in chronic hepatitis B associated with higher risk to hepatocarcinogenesis independent of presence of liver cirrhosis.

Reference	Factor	Location of Mutation
[42]	HBV serum level	
[45,50]	Genotype C	
[51]	Combined HbsAg/HbeAg positivity	
[52,54]	T1762/A1764 mutation	Basal core promoter
[46,55]	C1653T/T1753V mutation	Hbx
[61,62]	K130M/V131I/V131I mutation	Hbx
[66]	PreS deletion	PreS

## Data Availability

Not applicable.
